# Association Between Fresh Gas Flow Rate and Optic Nerve Sheath Diameter During Laparoscopic Cholecystectomy: A Prospective Observational Study

**DOI:** 10.3390/jcm15197626

**Published:** 2026-10-01

**Authors:** Mervenur Gizem Öztürk, Öznur Demiroluk, Mehmet Timuçin Aydın, Arzu Yıldırım Ar

**Affiliations:** 1 Department of Anesthesiology and Reanimation, Fatih Sultan Mehmet Training and Research Hospital, University of Health Sciences, Istanbul 34752, Türkiye; 2 Department of Anesthesiology and Reanimation, Karakoçan State Hospital, Elazığ 23600, Türkiye; 3 Department of General Surgery, Fatih Sultan Mehmet Training and Research Hospital, University of Health Sciences, Istanbul 34752, Türkiye

**Keywords:** desflurane, fresh gas flow, intracranial pressure, laparoscopic cholecystectomy, low-flow anesthesia, optic nerve sheath diameter

## Abstract

**Background/Objectives:** Carbon dioxide pneumoperitoneum during laparoscopic surgery may alter intracranial dynamics, while the influence of fresh gas flow on these changes remains incompletely understood. This prospective observational study investigated the association between fresh gas flow rate and perioperative optic nerve sheath diameter (ONSD) trajectories during desflurane anesthesia for laparoscopic cholecystectomy. **Methods:** Sixty-four adults undergoing elective laparoscopic cholecystectomy were classified according to the fresh gas flow rate received during routine anesthetic management as low flow (1 L/min; *n* = 32) or high flow (3 L/min; *n* = 32). Bilateral ONSD was measured ultrasonographically at six predefined time points (T0–T5) and analyzed using linear mixed-effects models. **Results:** ONSD changed significantly over time (*p* < 0.001), increasing after pneumoperitoneum and decreasing after desufflation. No significant overall group effect (*p* = 0.337) or group × time interaction (*p* = 0.356) was detected, and between-group contrasts were nonsignificant at all individual time points after Holm correction. Covariate-adjusted sensitivity analysis yielded consistent findings. No significant associations between ONSD and regional cerebral oxygen saturation were detected after Holm correction. Minimum alveolar concentration (MAC) showed a significant group × time interaction, with higher values in the high-flow group at T2 and T3, whereas bispectral index (BIS) showed no significant group or group × time effect. **Conclusions:** Under the studied conditions, no statistically significant difference in perioperative ONSD trajectories was detected between fresh gas flow rates of 1 L/min and 3 L/min during laparoscopic cholecystectomy. These findings provide prospective observational data on the relationship between fresh gas flow and perioperative ONSD but should not be interpreted as evidence of equivalence or physiological safety.

## 1. Introduction

Laparoscopic surgery is associated with physiological alterations resulting from carbon dioxide insufflation and increased intra-abdominal pressure, potentially leading to elevated intracranial pressure (ICP) [1,2,3]. Ultrasonographic measurement of optic nerve sheath diameter (ONSD) has gained increasing attention as a non-invasive surrogate for ICP because of its correlation with invasive ICP measurements [4,5,6].

Volatile anesthetic agents may influence cerebral blood flow and autoregulation, particularly at higher concentrations [7,8,9,10,11]. Desflurane has low blood–gas solubility and allows rapid adjustment of anesthetic concentration; however, its cerebral effects are also influenced by factors such as carbon dioxide tension and hemodynamic conditions.

Low-flow anesthesia offers economic and environmental advantages by reducing anesthetic gas consumption and atmospheric emissions [12,13,14]. However, the potential influence of fresh gas flow on cerebral physiology during laparoscopic procedures remains incompletely understood. Reduced fresh gas flow may alter carbon dioxide dynamics through increased rebreathing and may also affect volatile anesthetic delivery and kinetics. Even modest increases in arterial carbon dioxide tension can cause cerebral vasodilatation, increase cerebral blood flow, and potentially increase ICP. These considerations may be particularly relevant during laparoscopic surgery, where pneumoperitoneum and patient positioning can independently influence cerebral hemodynamics [3,15,16]. Whether different fresh gas flow rates are associated with clinically meaningful differences in perioperative intracranial dynamics remains uncertain.

This study primarily investigated the association between fresh gas flow rate and perioperative ONSD trajectory during desflurane anesthesia for laparoscopic cholecystectomy. We compared ONSD changes between patients managed with fresh gas flow rates of 1 L/min and 3 L/min. Additionally, the association between ONSD and regional cerebral oxygen saturation measured by near-infrared spectroscopy (NIRS) was explored.

## 2. Materials and Methods

### 2.1. Study Design and Participants

This prospective observational study was approved by the Clinical Research Ethics Committee of Istanbul Fatih Sultan Mehmet Training and Research Hospital, University of Health Sciences (approval No. 2023/150; approval date: 12 October 2023) and was conducted at Istanbul Fatih Sultan Mehmet Training and Research Hospital between November 2023 and March 2024 in accordance with the Declaration of Helsinki. The study was reported in accordance with the Strengthening the Reporting of Observational Studies in Epidemiology (STROBE) guidelines. Written informed consent was obtained from all participants.

Patients aged 18–65 years with an American Society of Anesthesiologists (ASA) physical status of I–III who were scheduled for elective laparoscopic cholecystectomy were eligible for inclusion. Exclusion criteria comprised intracranial pathology, previous neurosurgery, glaucoma, body mass index (BMI) ≥ 35 kg/m^2^, severe systemic disease, or emergency surgery. Data were collected from 67 patients; three were excluded from the final analysis because they did not meet the prespecified eligibility criteria (BMI ≥ 35 kg/m^2^, *n* = 2; age > 65 years, *n* = 1), leaving 64 patients for analysis.

Fresh gas flow rate was determined as part of routine clinical practice by the attending anesthesiologist responsible for intraoperative anesthetic management, independently of the study team. The attending anesthesiologist varied across operating days, and patients were subsequently classified as low flow (1 L/min) or high flow (3 L/min) according to the fresh gas flow rate they had received. Individual attending anesthesiologists did not routinely adhere to a single fresh gas flow strategy, and neither 1 L/min nor 3 L/min was preferentially used by a particular anesthesiologist as a fixed practice. No fresh gas flow assignment, matching, or balancing of group sizes was performed by the study investigators; the resulting groups each comprised 32 patients.

### 2.2. Anesthesia Protocol and Intraoperative Monitoring

Standard monitoring included electrocardiography (ECG), non-invasive blood pressure, peripheral oxygen saturation (SpO_2_), temperature, bispectral index (BIS; Covidien, Mansfield, MA, USA), and near-infrared spectroscopy (NIRS; INVOS™ 5100C Cerebral/Somatic Oximeter, Covidien, Mansfield, MA, USA). Anesthesia was induced with intravenous propofol (2–2.5 mg/kg), fentanyl (2 µg/kg), and rocuronium (0.6 mg/kg). After tracheal intubation, mechanical ventilation was standardized using a tidal volume of 6–8 mL/kg, with the respiratory rate adjusted to maintain end-tidal carbon dioxide (ETCO_2_) within the predefined target range. ETCO_2_ was continuously monitored by capnography using the anesthesia workstation. Inspired CO_2_ was also continuously monitored throughout anesthesia, and no clinically relevant CO_2_ rebreathing was observed. NIRS measurements were recorded at predefined intraoperative time points (T0–T4) corresponding to ONSD assessments.

Anesthesia was maintained with desflurane (4–6%) in a 50% O_2_–air mixture and continuous remifentanil infusion (0.05–0.2 µg/kg/min). Anesthetic agents were titrated to maintain BIS between 40 and 60, heart rate and mean arterial pressure within 20% of baseline values, and NIRS values within 20% of baseline measurements. During the initial wash-in period, a fresh gas flow of 4 L/min was used for approximately 4–8 min to achieve denitrogenation and establish the desired anesthetic gas concentration. Thereafter, fresh gas flow was reduced to 1 L/min or 3 L/min according to the routine clinical practice of the attending anesthesiologist. Minimum alveolar concentration (MAC) was recorded as the age-adjusted MAC value displayed by the anesthesia workstation at each predefined measurement time point.

Standard surgical positioning for laparoscopic cholecystectomy was applied in all patients. Patients were initially placed in the Trendelenburg position during establishment of CO_2_ pneumoperitoneum and subsequently transitioned to the reverse Trendelenburg position for the laparoscopic surgical period. After desufflation, patients were returned to the supine position. Intra-abdominal pressure was maintained at 12 mmHg during pneumoperitoneum. Pneumoperitoneum duration was defined as the interval from CO_2_ insufflation to desufflation and was recorded for each patient.

### 2.3. Optic Nerve Sheath Diameter Measurements

Optic nerve sheath diameter measurements were obtained through the closed eyelid using an ultrasound system (MyLab 30 Gold, Esaote, Genoa, Italy) equipped with a 12-MHz linear transducer. Measurements were performed perpendicular to the retina using electronic calipers at 3 mm posterior to the point where the optic nerve entered the globe. For each eye, two consecutive measurements were performed, and their mean was recorded. For the primary patient-level longitudinal analysis, left- and right-eye ONSD measurements were averaged at each time point to provide a single bilateral ONSD value.

Measurements were recorded at the following predefined time points: baseline (T0), post-intubation (T1), 5 min after pneumoperitoneum (T2), 20 min after pneumoperitoneum (T3), after desufflation (T4), and 15 min postoperatively in the recovery unit (T5). All ONSD measurements were performed by the same investigator to minimize inter-operator variability. The ONSD assessor was not involved in fresh gas flow selection or intraoperative anesthetic management and remained unaware of the applied fresh gas flow rate during each assessment.

### 2.4. Statistical Analysis

The a priori sample-size calculation was performed using G*Power version 3.1.9.7 based on the between-group ONSD data reported by Kim et al. [17]. A standardized effect size of Cohen’s *d* = 1.008, with a two-sided α of 0.05 and 90% power, indicated a minimum required sample size of 22 patients per group. Continuous variables are presented as mean (standard deviation [SD]) and categorical variables as the number (percentage). Baseline between-group characteristics were compared using the independent-samples *t*-test or Mann–Whitney U test for continuous variables and the chi-square test or Fisher’s exact test for categorical variables, as appropriate. Absolute standardized mean differences (SMDs) were additionally calculated to describe baseline between-group balance.

The primary outcome was the longitudinal trajectory of bilateral mean ONSD across the predefined perioperative measurement time points (T0–T5). The primary hypothesis was that this trajectory would differ between the 1 L/min and 3 L/min fresh gas flow groups, as assessed by the group × time interaction. Longitudinal ONSD was analyzed using a linear mixed-effects model with a participant-specific random intercept and fixed effects for fresh gas flow group, time as a categorical variable, and the group × time interaction. Bilateral ONSD measurements were averaged at each time point for the primary patient-level longitudinal analysis. Separate left- and right-eye mixed-effects models were performed as sensitivity analyses. A covariate-adjusted sensitivity model additionally included age, sex, body mass index, and ASA physical status. Omnibus fixed effects were evaluated using Wald chi-square tests. Model-based between-group contrasts at individual time points were adjusted for multiple comparisons using the Holm method.

Secondary repeated intraoperative variables, including mean arterial pressure, end-tidal carbon dioxide, bispectral index, and minimum alveolar concentration, were analyzed using analogous linear mixed-effects models with a participant-specific random intercept and fixed effects for fresh gas flow group, time as a categorical variable, and the group × time interaction. To account for multiplicity across these secondary outcomes, Holm correction was applied to the group × time interaction *p*-values. Associations between ONSD and regional cerebral oxygen saturation measured by near-infrared spectroscopy were assessed using Spearman’s rank correlation coefficients, with Holm adjustment for multiple testing. Statistical analyses were performed using IBM SPSS Statistics version 25.0 (IBM Corp., Armonk, NY, USA) and Python version 3.13.5 with the statsmodels package version 0.14.6. All tests were two-sided, and *p* < 0.05 was considered statistically significant.

## 3. Results

Data were collected from 67 patients; three were excluded from the final analysis because they did not meet the prespecified eligibility criteria (BMI ≥ 35 kg/m^2^, *n* = 2; age > 65 years, *n* = 1), leaving 64 patients for analysis, with 32 patients in each group. Complete ONSD measurements were available for all 64 analyzed patients at all predefined measurement time points (T0–T5), with no missing ONSD data in the primary longitudinal analysis. Baseline demographic and clinical characteristics are presented in Table 1. Although no statistically significant between-group differences were identified in the baseline characteristics, standardized mean differences indicated some imbalance in ASA physical status and comorbidity-related characteristics.

Longitudinal analyses of intraoperative physiological and anesthetic variables are presented in Table 2. MAP, ETCO_2_, and BIS changed significantly over time (all *p* < 0.001), but no significant overall group effects or group × time interactions were detected for these variables. MAC also changed significantly over time (*p* < 0.001), with no significant overall group effect (*p* = 0.181); however, the group × time interaction was significant after correction for multiple testing (Holm-adjusted *p* = 0.002). Subsequent model-based post-hoc contrasts showed higher MAC values in the high-flow group at T2 (Holm-adjusted *p* = 0.025) and T3 (Holm-adjusted *p* = 0.039), whereas between-group differences at T1 and T4 were not significant.

ONSD changed significantly over time (*p* < 0.001), whereas no significant overall group effect (*p* = 0.337) or group × time interaction (*p* = 0.356) was detected. Model-based between-group contrasts at individual time points showed no significant differences between the low- and high-flow groups after Holm adjustment (all adjusted *p* = 1.000). The estimated between-group differences were small across all measurement time points, with 95% confidence intervals including zero (Table 3). The covariate-adjusted sensitivity analysis yielded consistent findings, with no significant overall group effect (*p* = 0.336) or group × time interaction (*p* = 0.356).

Separate left- and right-eye sensitivity analyses yielded findings consistent with the primary bilateral ONSD analysis, with no significant group × time interactions (Supplementary Table S1).

No significant associations were detected between ONSD and regional cerebral oxygen saturation at any measurement time point in either group after Holm adjustment (all adjusted *p* ≥ 0.730; Supplementary Table S2).

## 4. Discussion

In this prospective observational study, we evaluated ONSD changes in patients receiving fresh gas flow rates of 1 L/min and 3 L/min during desflurane anesthesia for laparoscopic cholecystectomy. ONSD changed significantly over time, increasing after pneumoperitoneum and subsequently decreasing after desufflation. However, no significant overall group effect or group × time interaction was detected, and model-based between-group contrasts showed no significant differences at individual time points after adjustment for multiple comparisons. These findings indicate that no statistically significant between-group differences in the perioperative ONSD trajectory were detected under the conditions of this study. Because fresh gas flow selection was determined by routine clinical practice rather than randomization, the findings should be interpreted as observational associations rather than evidence of equivalence or causality.

Laparoscopic procedures are known to influence intracranial dynamics through carbon dioxide insufflation and increased intra-abdominal pressure, potentially impairing venous return and increasing intracranial pressure (ICP) [1,2,3,18,19]. Ultrasonographic ONSD measurement correlates with invasive ICP monitoring and has gained acceptance as a non-invasive surrogate in perioperative settings [4,5,6,20,21]. Although invasive ICP monitoring remains the gold standard, it is associated with procedural risks such as infection and hemorrhage [21]. A recent meta-analysis confirmed the reliability of ONSD as a non-invasive estimator of elevated ICP across diverse clinical contexts [6]. Consistent with previous reports, our findings showed transient increases in ONSD after pneumoperitoneum in both fresh gas flow groups.

Carbon dioxide pneumoperitoneum increases intra-abdominal pressure and may elevate intrathoracic pressure, thereby reducing cerebral venous outflow through increased central venous pressure [1,2,3,18,19]. In addition, systemic absorption of carbon dioxide can lead to cerebral vasodilatation, particularly in the presence of even mild hypercapnia, further contributing to transient elevations in intracranial pressure. Although ETCO_2_ was controlled throughout the procedure, the combined effects of pneumoperitoneum, positioning, and altered venous return may have contributed to the observed increases in ONSD following insufflation. Mechanical and hemodynamic factors may therefore play an important role in transient ICP-related changes during laparoscopic surgery. Accordingly, any potential effect of fresh gas flow may have been difficult to distinguish under controlled perioperative conditions. This may partly explain the inconsistent findings reported in previous studies regarding the impact of fresh gas flow on intracranial dynamics.

Desflurane is characterized by low blood–gas solubility and rapid titratability [7,8,9]. While volatile agents may influence cerebral blood flow and autoregulation at higher concentrations [10,11], their intracranial effects are generally more predictable when ventilation and hemodynamic conditions are controlled. In the present study, MAP, ETCO_2_, and BIS were closely controlled throughout the procedure. Control of carbon dioxide was particularly relevant because arterial carbon dioxide tension is a potent modulator of cerebral blood flow, and even mild hypercapnia may induce cerebral vasodilatation and increase intracranial pressure. However, ETCO_2_ is an indirect estimate of arterial carbon dioxide tension (PaCO_2_) and may not accurately reflect PaCO_2_ in all patients, particularly during pneumoperitoneum. Therefore, although the absence of a significant group × time interaction for ETCO_2_ supports broadly similar ventilatory trajectories between groups, unmeasured differences in arterial carbon dioxide tension cannot be excluded.

From a theoretical perspective, concerns regarding low-flow anesthesia include the potential for carbon dioxide rebreathing, altered volatile anesthetic kinetics, and fluctuations in inspired gas concentrations [12,13,14]. Modern anesthesia workstations allow close monitoring and control of ventilation and gas composition, which may mitigate these concerns under routine clinical conditions. In our cohort, the longitudinal trajectories of ETCO_2_ and hemodynamic variables did not differ significantly between fresh gas flow groups. Nevertheless, residual confounding related to clinical practice patterns cannot be excluded because fresh gas flow was not randomly assigned.

Mermer and Kozanhan reported lower ONSD values in a low-flow desflurane group than in a normal-flow group during laparoscopic cholecystectomy [22]. In contrast, our longitudinal analysis showed a significant change in ONSD over time but no significant overall group effect or group × time interaction. Furthermore, model-based between-group contrasts at individual time points remained nonsignificant after adjustment for multiple comparisons. An important methodological difference between the studies is the fresh gas flow range applied: Mermer and Kozanhan defined low- and normal-flow anesthesia as 0.75–1.5 L/min, whereas our study compared fixed flow rates of 1 L/min and 3 L/min. Differences in flow exposure, perioperative management, and study design may therefore contribute to the differing findings. In our cohort, ONSD values remained below commonly cited thresholds associated with elevated intracranial pressure [4,5,6,20]. However, the absence of a significant group × time interaction should not be interpreted as evidence of equivalence between flow strategies. Larger randomized studies with prespecified longitudinal analyses are needed to determine whether fresh gas flow independently influences perioperative ONSD trajectories.

A recent comparative study of sevoflurane and desflurane during laparoscopic cholecystectomy similarly reported no significant between-group differences in ONSD [23]. Although that study compared volatile anesthetic agents rather than fresh gas flow strategies, its findings provide additional context for interpreting perioperative ONSD changes during laparoscopic surgery.

A significant group × time interaction was observed for MAC, with higher MAC values in the high-flow group at T2 and T3, whereas BIS showed no significant group or group × time effect. This dissociation suggests that the observed MAC differences did not translate into detectable differences in hypnotic depth as assessed by BIS. The lower MAC values observed with lower fresh gas flow may reflect differences in volatile anesthetic delivery and equilibration after transition from the initial wash-in period to the maintenance flow rates. Previous studies have demonstrated that fresh gas flow influences desflurane kinetics under low-flow conditions [8,9]. Importantly, anesthetic management in the present study included remifentanil infusion in addition to desflurane, with anesthetic administration titrated to maintain BIS and hemodynamic variables within predefined target ranges. Therefore, the transient differences in MAC at T2 and T3 should be interpreted in the context of the overall anesthetic regimen and comparable BIS values, rather than as evidence of inadequate anesthetic depth in the low-flow group.

No significant associations were detected between ONSD and regional cerebral oxygen saturation measured by NIRS at any evaluated time point after adjustment for multiple comparisons. ONSD and NIRS, however, reflect distinct aspects of cerebral physiology: ONSD serves as a surrogate marker of intracranial pressure-related changes, whereas NIRS provides an estimate of regional cerebral oxygen saturation [24,25]. These modalities should therefore be regarded as complementary rather than interchangeable monitoring tools. The absence of a significant association in our cohort may reflect these physiological differences and should not be interpreted as evidence that changes in intracranial dynamics are unrelated to cerebral oxygenation.

From a clinical perspective, no statistically significant between-group difference in ONSD trajectory was detected under the controlled perioperative conditions of this study. Although concerns regarding low-flow anesthesia include potential effects related to carbon dioxide rebreathing, volatile anesthetic kinetics, and cerebral hemodynamics, no significant association between fresh gas flow strategy and the perioperative ONSD trajectory was detected in this cohort. However, because flow strategy was determined by routine clinical practice rather than randomization, causal inference cannot be established, and these findings should not be interpreted as evidence of equivalence or definitive physiological safety. Rather, these findings add longitudinal clinical evidence to the limited literature examining the relationship between fresh gas flow and intracranial dynamics during laparoscopic surgery and provide a basis for future randomized studies.

### Limitations

This study has several limitations. First, the non-randomized observational design may have introduced selection bias and residual confounding, because fresh gas flow was determined according to routine clinical practice rather than investigator-directed allocation. Although baseline balance was assessed and covariate-adjusted sensitivity analyses were performed, unmeasured confounding cannot be excluded; therefore, the independent contribution of fresh gas flow to ONSD changes cannot be definitively established.

Second, although the achieved sample size exceeded the prespecified requirement, the original sample-size calculation was based on a between-group ONSD effect and was not specifically designed for the longitudinal group-by-time interaction; therefore, smaller or transient interaction effects may have remained undetected.

Third, the study was conducted at a single center and included relatively low-risk patients undergoing elective laparoscopic cholecystectomy; patients with intracranial pathology, severe obesity (BMI ≥ 35 kg/m^2^), or severe systemic disease were excluded. These selection criteria limit the external validity of the findings, which may not be generalizable to higher-risk populations, patients with pre-existing intracranial pathology, or other surgical settings.

Fourth, although ventilation was standardized and ETCO_2_ was continuously monitored and maintained within the predefined target range, arterial blood gas analysis was not performed. Therefore, PaCO_2_ was not directly measured, and potential variations in arterial CO_2_ that may have influenced cerebral vascular tone and ONSD cannot be completely excluded. Future studies incorporating simultaneous PaCO_2_ measurements may help further characterize the relationship between fresh gas flow, CO_2_ homeostasis, and perioperative ONSD changes.

Fifth, ONSD is a clinically useful, non-invasive surrogate marker for assessing changes related to intracranial pressure; however, it does not constitute a direct measurement of ICP, and invasive ICP monitoring was not performed in this study. Accordingly, the absence of a significant between-group difference in ONSD suggests comparable ONSD trajectories under the studied conditions but should not be interpreted as proof of equivalent intracranial physiological effects or as direct evidence of physiological safety.

Finally, ONSD measurements were limited to the intraoperative and early postoperative period, precluding the assessment of later postoperative changes. Larger randomized studies including higher-risk populations, direct assessment of arterial carbon dioxide tension, and longer postoperative follow-up are warranted.

## 5. Conclusions

During laparoscopic cholecystectomy, perioperative ONSD changed significantly over time, whereas no statistically significant difference in ONSD trajectories was detected between patients receiving fresh gas flow rates of 1 L/min and 3 L/min. The observed ONSD changes appeared to follow the perioperative time course, including pneumoperitoneum and desufflation. These findings provide prospective observational data on the relationship between fresh gas flow and perioperative ONSD; however, given the non-randomized design and the use of ONSD as a surrogate marker of intracranial pressure, they should not be interpreted as evidence of equivalence, causality, or physiological safety. Larger randomized studies are warranted to further determine whether fresh gas flow independently influences perioperative ONSD trajectories.

## Figures and Tables

**Table 1:** Demographic and baseline characteristics of the study groups.

Variable	Low Flow (1 L/min) (*n* = 32)	High Flow (3 L/min) (*n* = 32)	*p*-Value	SMD
Sex, *n* (%)			1.000 ^1^	
Female	20 (62.5)	21 (65.6)		0.07
Male	12 (37.5)	11 (34.4)		
ASA physical status, *n* (%)			0.243 ^2^	
I	8 (25.0)	3 (9.4)		0.42
II	20 (62.5)	25 (78.1)		0.35
III	4 (12.5)	4 (12.5)		0.00
Age (years)	44.3 (11.4)	47.4 (10.0)	0.216 ^3^	0.29
Height (cm)	169.5 (7.8)	168.2 (8.8)	0.396 ^3^	0.16
Weight (kg)	79.9 (10.3)	78.4 (13.5)	0.612 ^4^	0.13
BMI (kg/m^2^)	27.4 (2.8)	27.2 (3.3)	0.776 ^4^	0.07
Pneumoperitoneum duration (min)	60.6 (21.5)	56.8 (29.0)	0.220 ^3^	0.15
Anesthesia duration (min)	74.5 (21.6)	70.0 (29.4)	0.176 ^3^	0.17
Comorbidities, *n* (%)				
≥2 comorbidities	6 (18.7)	11 (34.4)	0.257 ^1^	0.36
Hypertension	5 (15.6)	11 (34.4)	0.148 ^1^	0.44
Diabetes mellitus	4 (12.5)	6 (18.8)	0.732 ^1^	0.17
Smoking	10 (31.3)	9 (28.1)	1.000 ^1^	0.07
Respiratory disease	2 (6.3)	5 (15.6)	0.426 ^1^	0.30

Data are presented as mean (SD) or *n* (%). *p*-values were obtained using ^1^ Fisher’s exact test, ^2^ the Pearson chi-square test, ^3^ the Mann–Whitney U test, or ^4^ the independent-samples *t*-test, as indicated. Absolute standardized mean differences (SMDs) are provided to describe the magnitude of between-group differences in baseline characteristics. SMDs for categorical variables are presented for the indicated category. All *p*-values are two-sided, and *p* < 0.05 was considered statistically significant. **Abbreviations:** ASA, American Society of Anesthesiologists; BMI, body mass index; SD, standard deviation; SMD, standardized mean difference.

**Table 2:** Intraoperative physiological and anesthetic variables and longitudinal mixed-effects model results.

Variable	Time	Low Flow (1 L/min) (*n* = 32)	High Flow (3 L/min) (*n* = 32)	*p* for Group	*p* for Time	*p* for Group × Time	Holm-Adjusted *p* for Interaction *
MAP (mmHg)	T0	102.3 (11.6)	104.9 (9.3)	0.189	<0.001	0.608	1.000
	T1	84.8 (12.2)	91.1 (12.0)				
	T2	95.5 (11.2)	97.6 (15.5)				
	T3	91.1 (9.9)	91.2 (15.3)				
	T4	86.5 (9.1)	89.4 (13.8)				
	T5	100.0 (10.2)	101.5 (11.3)				
ETCO_2_ (mmHg)	T1	31.3 (2.5)	30.7 (2.9)	0.127	<0.001	0.846	1.000
	T2	33.3 (2.8)	32.5 (3.0)				
	T3	34.5 (2.5)	33.8 (2.9)				
	T4	34.5 (2.6)	33.4 (2.4)				
BIS	T0	91.8 (3.1)	92.0 (3.3)	0.844	<0.001	0.705	1.000
	T1	43.3 (7.0)	44.2 (7.2)				
	T2	42.4 (6.2)	41.5 (5.7)				
	T3	42.6 (5.6)	42.5 (5.7)				
	T4	45.3 (6.4)	46.4 (6.7)				
MAC	T1	0.64 (0.09)	0.64 (0.12)	0.181	<0.001	<0.001	0.002
	T2 †	0.66 (0.06)	0.73 (0.09)				
	T3 †	0.68 (0.06)	0.74 (0.10)				
	T4	0.64 (0.10)	0.62 (0.16)				

Data are presented as mean (SD). Longitudinal variables were analyzed using linear mixed-effects models with a participant-specific random intercept and fixed effects for fresh gas flow group, time (categorical), and the group × time interaction. *p*-values for group, time, and group × time represent omnibus Wald chi-square tests and therefore apply to the complete longitudinal trajectory of each variable rather than to individual time points. * Holm-adjusted *p*-values account for multiple testing across the four secondary group × time interaction tests. † For MAC, the significant group × time interaction was followed by model-based between-group contrasts at individual time points. After Holm adjustment, MAC was significantly higher in the high-flow group at T2 (*p* = 0.025) and T3 (*p* = 0.039); between-group contrasts at T1 and T4 were not significant. All *p*-values are two-sided, and *p* < 0.05 was considered statistically significant. **Abbreviations:** BIS, bispectral index; ETCO_2_, end-tidal carbon dioxide; MAC, minimum alveolar concentration; MAP, mean arterial pressure; SD, standard deviation. T0, baseline; T1, post-intubation; T2, 5 min after pneumoperitoneum; T3, 20 min after pneumoperitoneum; T4, after desufflation; T5, 15 min postoperatively.

**Table 3:** Optic nerve sheath diameter measurements and longitudinal mixed-effects model results.

Time	Low Flow (1 L/min) (*n* = 32)	High Flow (3 L/min) (*n* = 32)	Mean Difference (95% CI) *	Holm-Adjusted *p* *
T0	5.117 (0.309)	5.227 (0.392)	−0.109 (−0.273 to 0.055)	1.000
T1	5.370 (0.313)	5.464 (0.376)	−0.094 (−0.258 to 0.070)	1.000
T2	5.544 (0.307)	5.625 (0.382)	−0.081 (−0.245 to 0.083)	1.000
T3	5.656 (0.294)	5.714 (0.417)	−0.058 (−0.222 to 0.106)	1.000
T4	5.395 (0.263)	5.481 (0.370)	−0.086 (−0.250 to 0.078)	1.000
T5	5.227 (0.273)	5.263 (0.344)	−0.036 (−0.200 to 0.128)	1.000
Longitudinal mixed-effects model				*p*-value
Group				0.337
Time				<0.001
Group × time				0.356

Data are presented as mean (SD) in mm. Bilateral ONSD measurements were averaged at each time point to provide a single patient-level value for the primary longitudinal analysis. ONSD was analyzed using a linear mixed-effects model with a participant-specific random intercept and fixed effects for fresh gas flow group, time (categorical), and the group × time interaction. *p*-values for group, time, and group × time represent omnibus Wald chi-square tests and apply to the complete longitudinal ONSD trajectory rather than to individual time points. * Mean differences represent model-based low-flow minus high-flow contrasts at each time point, with 95% confidence intervals. *p*-values for these time-specific between-group contrasts were adjusted for multiple comparisons using the Holm method. Separate left- and right-eye mixed-effects models were performed as sensitivity analyses. A covariate-adjusted sensitivity model additionally included age, sex, body mass index, and ASA physical status. All *p*-values are two-sided, and *p* < 0.05 was considered statistically significant. **Abbreviations:** ASA, American Society of Anesthesiologists; CI, confidence interval; ONSD, optic nerve sheath diameter; SD, standard deviation. T0, baseline; T1, post-intubation; T2, 5 min after pneumoperitoneum; T3, 20 min after pneumoperitoneum; T4, after desufflation; T5, 15 min postoperatively.

## Data Availability

The datasets generated and analyzed during the current study are available from the corresponding author on reasonable request.

## Supplementary Materials

The following supporting information can be downloaded at https://www.mdpi.com/article/10.3390/jcm15197626/s1. Table S1: Separate left- and right-eye sensitivity analyses of optic nerve sheath diameter; Table S2: Associations between optic nerve sheath diameter and regional cerebral oxygen saturation.
