# Supplementary material for: Association Between Fresh Gas Flow Rate and Optic Nerve Sheath Diameter During Laparoscopic Cholecystectomy: A Prospective Observational Study
Source: J Clin Med. 2026 Oct 1;15(19):7626. doi: 10.3390/jcm15197626 (PMC13666220; doi:10.3390/jcm15197626)
Supplement: Supplementary file 1 [file jcm-15-07626-s001.zip › jcm-4586673-supplementary.pdf]

Supplementary Table S1.

Separate left- and right-eye sensitivity analyses of optic nerve sheath diameter

Eye	Effect	Wald χ^2	df	P value
**Left**	Group	0.96	1	0.328
	Time	916.07	5	< 0.001
	Group × time	9.53	5	0.090
**Right**	Group	0.76	1	0.384
	Time	972.38	5	< 0.001
	Group × time	6.70	5	0.244

Separate left- and right-eye ONSD measurements were analyzed as sensitivity analyses using linear mixed-effects models with a participant-specific random intercept and fixed effects for fresh gas flow group, time (categorical), and the group × time interaction. P values represent omnibus Wald chi-square tests and apply to the complete longitudinal trajectory for each eye. These sensitivity analyses were consistent with the primary bilateral ONSD analysis, with significant changes over time but no significant overall group effects or group × time interactions. All P values are two-sided, and $P < 0.05$ was considered statistically significant.

Abbreviations: df, degrees of freedom; ONSD, optic nerve sheath diameter.

Supplementary Table S2.

Associations between optic nerve sheath diameter and regional cerebral oxygen saturation

Time	Low-flow Spearman's ρ	Low-flow Holm-adjusted P	High-flow Spearman's ρ	High-flow Holm-adjusted P
**T0**	−0.077	1.000	0.263	0.730
**T1**	0.181	1.000	0.194	1.000
**T2**	0.084	1.000	−0.097	1.000
**T3**	0.198	1.000	−0.056	1.000
**T4**	0.155	1.000	−0.190	1.000

Bilateral ONSD and NIRS measurements were averaged at each time point to provide single patient-level values. Associations between ONSD and regional cerebral oxygen saturation measured by NIRS were assessed using Spearman's rank correlation coefficient (ρ). P values were adjusted for multiple comparisons across the five time-point correlations within each group using the Holm method. All P values are two-sided, and $P < 0.05$ was considered statistically significant.

Abbreviations: NIRS, near-infrared spectroscopy; ONSD, optic nerve sheath diameter. T0, baseline; T1, post-intubation; T2, 5 min after pneumoperitoneum; T3, 20 min after pneumoperitoneum; T4, after desufflation.
